# Estradiol and GPER Activation Differentially Affect Cell Proliferation but Not GPER Expression in the Hippocampus of Adult Female Rats

**DOI:** 10.1371/journal.pone.0129880

**Published:** 2015-06-15

**Authors:** Paula Duarte-Guterman, Stephanie E. Lieblich, Carmen Chow, Liisa A. M. Galea

**Affiliations:** Department of Psychology, Program in Neuroscience, Centre for Brain Health, University of British Columbia, Vancouver, British Columbia, Canada; University of Lethbridge, CANADA

## Abstract

Estradiol increases cell proliferation in the dentate gyrus of the female rodent but it is not known whether the G protein-coupled estrogen receptor (GPER), a membrane receptor, is involved in this process, nor whether there are regional differences in estradiol’s effects on cell proliferation. Thus, we investigated whether estradiol exerts its effects on cell proliferation in the dorsal and ventral dentate gyrus through GPER, using the GPER agonist, G1, and antagonist, G15. Ovariectomized adult female rats received a single injection of either: 17β-estradiol (10 μg), G1 (0.1, 5, 10 μg), G15 (40 μg), G15 and estradiol, or vehicle (oil, DMSO, or oil+DMSO). After 30 min, animals received an injection of bromodeoxyuridine (BrdU) and were perfused 24 h later. Acute treatment with estradiol increased, while the GPER agonist G1 (5 μg) decreased, the number of BrdU+ cells in the dentate gyrus relative to controls. The GPER antagonist, G15 increased the number of BrdU+ cells relative to control in the dorsal region and decreased the number of BrdU+ cells in the ventral region. However, G15 treatment in conjunction with estradiol partially eliminated the estradiol-induced increase in cell proliferation in the dorsal dentate gyrus. Furthermore, G1 decreased the expression of GPER in the dentate gyrus but not the CA1 and CA3 regions of the hippocampus. In summary, we found that activation of GPER decreased cell proliferation and GPER expression in the dentate gyrus of young female rats, presenting a potential and novel estrogen-independent role for this receptor in the adult hippocampus.

## Introduction

Neurogenesis occurs throughout the lifespan in the mammalian dentate gyrus [1,2,3,4]. Estradiol influences hippocampal neurogenesis by modulating both cell proliferation and survival of young neurons in female rodents (reviewed in [5]). In ovariectomized young adult female rats, 17β-estradiol increases cell proliferation after 30 minutes and 2 hours of exposure, but not after 4 hours [6,7,8] and decreases cell proliferation after 48 h [9]. The fast-acting effects of estradiol (between 30 min and 2 h) suggest a possible non-genomic action to increase cell proliferation [10,11] while the longer effects (at 48 h) may involve genomic mechanisms via estradiol binding to nuclear estrogen receptors (ERα and ERβ) [11]. We previously found that administration of either an ERα or ERβ agonist (PPT and DPN, respectively) increases cell proliferation in adult ovariectomized rats; however, PPT and DPN, alone or in combination did not increase proliferation to the levels seen with estradiol [12]. In addition, the effects of estradiol are only partially blocked with the ER antagonist ICI 182,780 [13] suggesting that the modulation of cell proliferation by estradiol cannot be completely explained by the actions on these nuclear ERs and that an alternative mechanism(s) may be at work.

A G protein-coupled estrogen receptor (GPER, formerly GPR30) has been recognized as an estrogen receptor localized in the plasma membrane and endoplasmic reticulum (reviewed in [14]). GPER is expressed in the dentate gyrus, CA1, and CA3 regions of the hippocampus in adult male and female rodents [15,16,17]. However, it is not known whether activation of GPER or treatment with estradiol regulate GPER expression levels in the hippocampus and the present study served to address this gap in the literature. Treatment with the GPER agonist (G1) enhances hippocampus-dependent spatial memory similar to the effects of estradiol in female rats [18,19]. Alternatively, treatment with the GPER antagonist, G15, blocked the effect of estradiol on spatial memory [20] indicating that GPER mediates at least some of estradiol’s effects on hippocampus-dependent memory. These data collectively suggest a possible regulatory role of GPER on hippocampal function and adult neurogenesis. Curiously, even though estradiol, PPT, and DPN increase cell proliferation, few nuclear ERα or ERβ are co-localized with proliferating cells in the dentate gyrus [12]. Thus given the effects of estradiol to promote cell proliferation within hours, we also sought to determine whether GPER is expressed in proliferating cells in the dentate gyrus.

In this study, we investigated the role of GPER in regulating hippocampal cell proliferation in adult female rats. We used a GPER agonist (G1) and antagonist (G15) to determine whether GPER mediates the estradiol-induced increase in cell proliferation. We hypothesized that activation of GPER with G1 would increase cell proliferation similar to estradiol while G15 would reduce the estradiol-induced increase in cell proliferation. In addition, we investigated whether estradiol, G1, and G15 regulate the expression of GPER in the dentate gyrus, CA1, and CA3 regions of the hippocampus. Finally, we determined whether dividing cells in the dentate gyrus express GPER and if so, whether estradiol, G1, and G15 treatments influenced co-localization with progenitor cells.

## Materials and Methods

### Animals and surgery

Sixty-three adult female Sprague–Dawley rats (approximately 250 g) were obtained from Charles River (Quebec, Canada). The protocol was approved by the University of British Columbia Animal Care Committee and strictly followed the guidelines of the Canadian Council on Animal Care. Isoflurane was used as the form of anesthesia during surgeries and all efforts were made to minimize animal suffering. For euthanasia, rats were deeply anesthetized with sodium pentobarbital and then perfused with 0.9% saline followed by 4% paraformaldehyde. Rats were housed in pairs with aspen chip bedding, given food and water ad libitum, and maintained under a 12 h light/dark cycle starting at 0700. One week after arrival, rats were bilaterally ovariectomized using aseptic techniques under isoflurane (2–3%) and given lactated ringer’s solution (5ml; subcutaneously, s.c.), anafen (5mg/kg; s.c), and marcaine (4mg/kg; s.c. locally). Rats were single-housed and handled for 5 consecutive days post-surgery.

### Treatments

Rats were randomly assigned to a treatment group (n = 6–7/treatment). In Experiment 1, one week after surgery, rats received a single injection (0.1 ml) of either: sesame oil (s.c.); 17β-estradiol (10 μg in oil; s.c.), DMSO (70% in 0.9% saline, intraperitoneally, i.p.), or G1 (low, 0.1 μg, medium, 5 μg, or high, 10 μg, in 70% DMSO, i.p). In Experiment 2, a second cohort of rats received a combination of injections: DMSO (i.p.) and oil (s.c.), G15 (40 μg; i.p.) and oil, DMSO and estradiol (10 μg; s.c.), or G15 (40 μg; i.p.) and estradiol (10 μg; s.c.). The doses and routes of administration of estradiol, G1, and G15 were chosen based on previous studies [6,18,20,21]. The 10 μg dose of 17β-estradiol results in serum estradiol levels similar to proestrus and this dose increases apical spine density, cell proliferation, and lordosis and influences hippocampus-dependent memory [6,22,23,24,25]. Estradiol (Sigma-Aldrich) was prepared over low heat. G1 and G15 (R&D Systems, Minneapolis, MN, USA) were first dissolved in 100% DMSO and then diluted to 70% with 0.9% saline. G1 and G15 were dissolved in the lowest possible concentration of DMSO (i.e., 70%) and according to the manufacturer’s recommendation. In both experiments, 30 min after the injections, animals received a single injection of the DNA synthesis marker, bromodeoxyuridine (BrdU; 200 mg/kg; i.p; Sigma-Aldrich, Oakville, ON, Canada). BrdU was prepared by dissolving BrdU to 20 mg/ml in warm 0.9% saline buffered with 0.7% NaOH. BrdU is active for 2 h after the initial injection and is incorporated into any cell that is synthesizing DNA during that time window. The cell cycle in the hippocampus of adult male rats is approximately 24 h indicating that after 24 h, BrdU+ cells are daughter cells [26]. We chose 24 h in order to (1) examine daughter cells of proliferating labelled progenitors (labelled within a 2 h period after BrdU injection), (2) to match the previous literature [6,9,12,13], and (3) to minimize the effects of stress on cell proliferation by avoiding colony disruptions while BrdU was still active. Therefore, 24 h after the BrdU injection, rats were transcardially perfused with cold 0.9% saline and 4% paraformaldehyde (Sigma-Aldrich) and brains were dissected and post fixed in 4% paraformaldehyde for 24 h at 4°C, then transferred to 30% sucrose (Fisher Scientific, Ottawa, ON). Serial coronal sections (40 μm) were cut with a freezing microtome (SM2000R; Leica, Richmond Hill, ON) across the extent of the hippocampus (collected in 10 series) and stored in an antifreeze solution (containing ethylene glycol, glycerol and 0.1 M PBS) at -20°C.

### BrdU immunohistochemistry

Rinses and incubations were conducted on free-floating sections under moderate shaking at room temperature unless otherwise stated. Sections (series 1 of 10) were rinsed between steps with tris-buffered saline (0.1M TBS; pH = 7.4). Sections were first incubated in 0.8% H_2_O_2_ in TBS for 30 min to block endogenous peroxidase. To denature DNA, sections were then incubated in 2N HCl at 37°C and immediately transferred to 0.1M borate buffer (pH = 8.5) for 10 min. Sections were blocked with 3% normal horse serum (NHS) and 0.1% Triton X-100 in TBS for 30 min before incubation with primary antibody mouse monoclonal anti-BrdU (1:200 in TBS with 3% NHS and 0.1% Triton-X; Roche Diagnostics 11170376001; Laval, QC, Canada) for 22 h at 4°C as described previously [6,27,28]. Sections were then incubated with secondary biotinylated anti-mouse (1:200 in TBS with 3% NHS and 0.1% Triton-X; Vector Laboratories, Burlington, ON, Canada) for 4 h and in avidin-biotin complex solution for 1.5 h as described by the manufacturer (ABC kit, Vector Laboratories). Sections were stained with diaminobenzidine (Peroxidase Substrate Kit, Vector Laboratories) mounted onto slides and left to dry for 2 days. Sections were counter-stained with Cresyl Violet (0.2%, Fisher Scientific), dehydrated, cleared with xylene, and coverslipped with Permount (Fisher Scientific).

### GPER immunofluorescence

Free floating sections were rinsed between steps with phosphate buffered saline (PBS; 0.1M; pH = 7.4). Sections (series 2 of 10) were first blocked with 3% normal donkey serum (NDS) and 0.3% Triton-X in PBS for 40 min at room temperature and then incubated in polyclonal rabbit anti-GPER (1:250 in PBS with 3% NDS and 0.1% Triton-X; Lifespan Bioscience LS-A4272; Seattle, WA, USA) for 24 h at 4°C. Sections were then incubated with secondary antibody Cy3 donkey anti-rabbit (1:200 in PBS and 3% NDS; Jackson Immunoresearch, WestGrove, PA, USA) for 24 h at 4°C. Sections were mounted on slides and coverslipped with PVA-DABCO. Specificity of the GPER antibody has been confirmed in rat tissue (e.g., [29,30]). We further optimized the dilutions and controlled for non-specific labelling by incubating sections with PBS instead of the primary antibody. All the negative controls resulted in the absence of immunoreactivity.

### GPER/Ki67 double-labelling immunofluorescence

To determine whether proliferating cells express GPER, we used double-label immunofluorescence for GPER and Ki67. Ki67 is an endogenous marker of cell proliferation, expressed in the nucleus during the cell cycle except for G_0_ and the initial part of G_1_ [31] and because of this, Ki67 labels many more proliferating cells than BrdU [32]. Sections were rinsed between steps with PBS (0.1M; pH = 7.4) and blocked as previously described for GPER immunofluorescence. Sections (series 3 of 10) were incubated in mouse anti-Ki67 (1:200; Novocastra NCL-Ki67-MM1, Leica Biosystems) and polyclonal rabbit anti-GPR30 (1:250) diluted in PBS containing 3% NDS and 0.1% Triton-X for 24 h at 4°C. Sections were then incubated with secondary antibodies Cy3 donkey anti-mouse (1:200; Jackson Immunoresearch) and Alexa 488 donkey anti-rabbit (1:200; Life Technologies) diluted in PBS and 3% NDS for 24 h at 4°C. Sections were mounted on slides and coverslipped with PVA-DABCO. The Ki67 antibody has been characterized in rat brain tissue [32,33]. Here we optimized the dilutions and conditions for optimal double labelling with GPER. For negative controls, sections were incubated with PBS instead of the primary antibodies (omitting one antibody at a time). All the negative controls resulted in the absence of immunoreactivity.

### Data analyses

**Fig 1:**
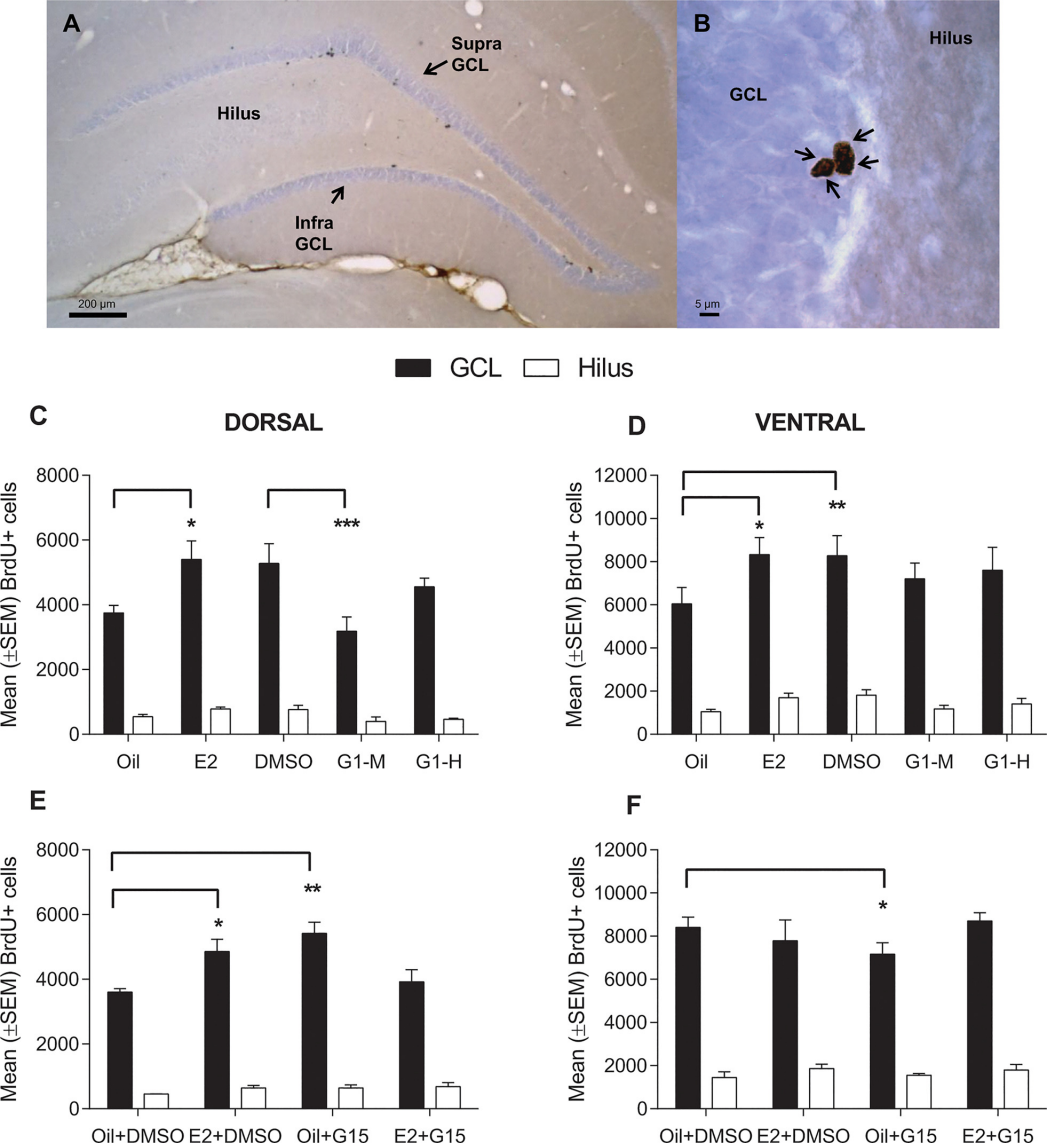
Estradiol, GPER agonist G1, and GPER antagonist G15 affect cell proliferation in the hippocampus. A photomicrograph of a representative section of (A) the dorsal dentate gyrus with the supra- and infra-pyramidal blades labelled and (B) cluster of BrdU+ cells (black arrows) in the subgranular zone of the dentate gyrus 24 h after BrdU injection. Mean (+SEM) total number of BrdU+ cells in the granule cell layer (GCL) and hilus of the dorsal (C and E) and ventral (D and F) dentate gyrus. For Experiment 1 (C-D), rats were given either estradiol (E2, 10 μg), one of the doses of G1 (M, medium, 5 μg; H, high, 10 μg) or one of the vehicles (oil or DMSO). Estradiol significantly increased cell proliferation in both dorsal and ventral regions, while G1-M decreased cell proliferation in the dorsal region only. DMSO alone significantly increased the number of BrdU+ cells in the ventral, but not dorsal GCL, compared to the oil group (P = 0.003, P = 0.021 respectively; dorsal not significant due to Bonferroni correction). For Experiment 2 (E-F), rats were given either estradiol (E2, 10 μg) or G15 (40 μg) alone or in combination with E2. Vehicle was a combination of oil and DMSO. E2+DMSO increased cell proliferation compared to control (oil+DMSO) in the dorsal GCL. G15 increased the number of BrdU+ cells relative to control in the dorsal region and decreased the number of BrdU+ cells in the ventral region compared to oil+DMSO. Asterisks denote significant differences between control and treatment groups (*p<0.05; **p<0.01; ***p<0.001).

**Fig 2:**
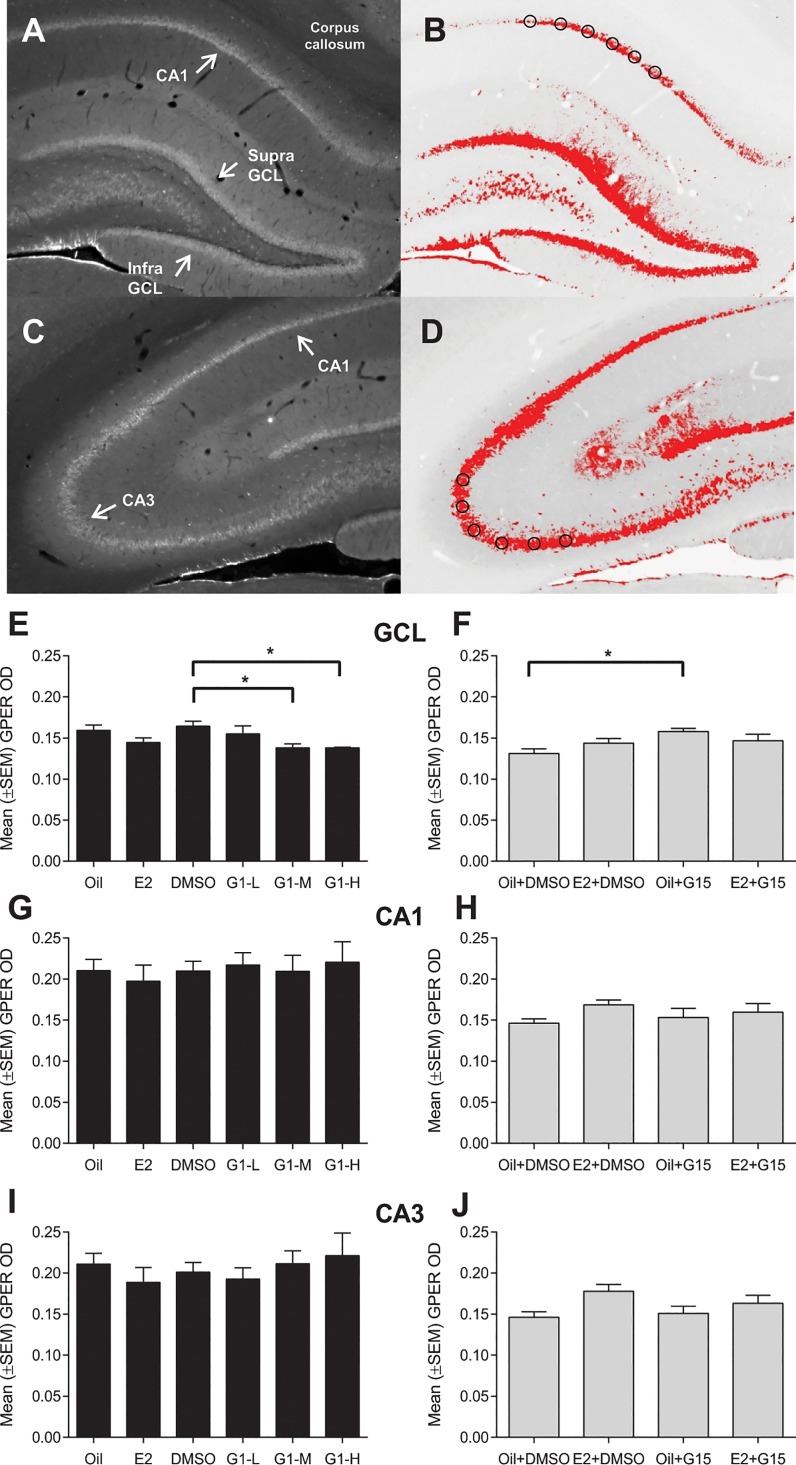
G1 and G15 regulate expression of GPER in the granule cell layer (GCL) but not the CA1 and CA3 regions of the hippocampus. Optical density (OD) was measured in the entire GCL, and in the CA1 and CA3 regions (using circles as shown in B and D). Photomicrograph of a representative section showing GPER expression in the GCL, CA1, and hilus (A) and the CA1 and CA3 regions (C). Thesholded images showing regions expressing GPER above the threshold (B and D). Mean (+SEM) optical density after 24 h of estradiol, G1 or G15 treatments in the total GCL including the subgranular zone (E-F), in the total CA1 (G-H) and total CA3 (I-J) regions. Rats were given either estradiol (E2, 10 μg), one of the doses of G1 (L, low, 0.1 μg; M, medium, 5 μg; H, high, 10 μg) or G15 (40 μg) alone or in combination with E2. Vehicles were either oil, DMSO, or a combination of both (oil+DMSO). Asterisks denote significant differences between treatment groups (*p<0.05; **p<0.01).

**Fig 3:**
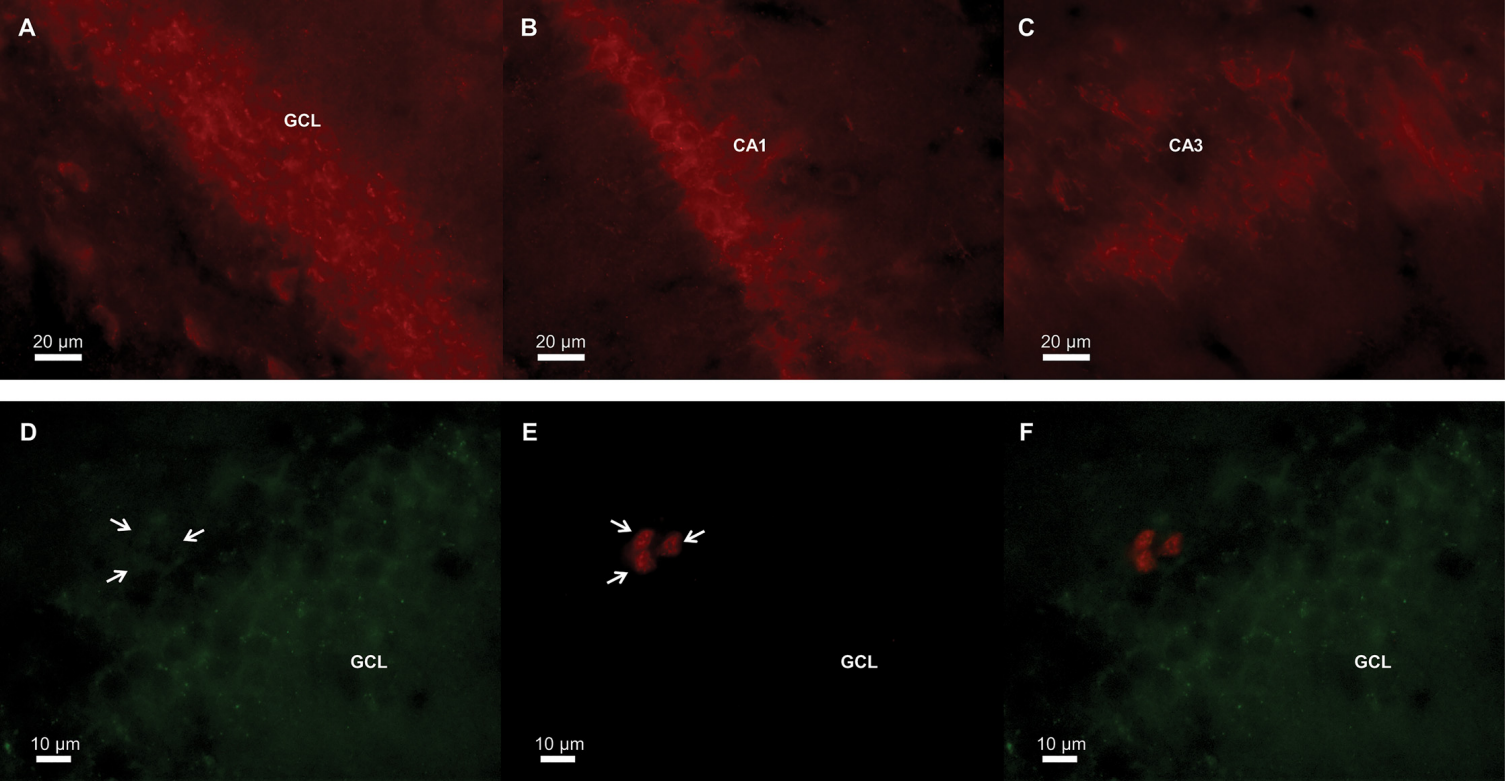
Photomicrographs of representative GPER expressing cells in the granule cell layer of the dentate gyrus (A), and the CA1 (B) and CA3 (C) regions of the hippocampus. GPER (green) is expressed in the granule cells (D) but not in proliferating (E) cells (red; arrows indicate Ki67-expressing cells), in the granule cell layer (GCL) of the dentate gyrus (F, merged figure).

An experimenter blind to the treatment groups counted BrdU+ cells in the granule cell layer (GCL) which included the subgranular zone (defined as approximately the 50 μm band between the GCL and the hilus) and hilus of the entire dentate gyrus (Fig 1A and 1B) at 1000X magnification (Olympus BX51 light microscope). Twenty-four hours after injection, BrdU+ cells are typically dark brown (intensely stained) and seen in clusters such as shown in Fig 1B [6,12,26]. Total BrdU+ cell counts were calculated by multiplying by 10 (to account for the fact that we used 1/10 series of sections–approximately 12–15 sections per rat) as previously described (e.g., [6,7]). GCL and hilus areas were calculated using ImageJ and volumes were calculated using Cavalieri’s principle [34]. We found dense expression of GPER in the GCL and CA regions (Figs 2 and 3) which could not be manually counted, therefore we assessed expression of GPER by quantitative densiometric analysis using ImageJ (Rasband, W.S., ImageJ, U. S. National Institutes of Health, Bethesda, Maryland, USA, http://imagej.nih.gov/ij/, 1997–2012). Photomicrographs of two dorsal and two ventral sections (both hemispheres, 4 sections total) per animal were taken at 40X magnification using the same exposure and gain settings. Optical density (OD) was assessed in the entire GCL including the subgranular zone. In the CA1 and CA3 regions, OD measurements were taken by placing six circles (each 80 μm in diameter) as shown in Fig 2B and 2D. OD measurements were adjusted automatically for background levels for each section by placing six circles within the corpus callosum of each section. Threshold levels were 1.75x above background levels. Measurements of the 6 circles (in CA1 and CA3) or entire GCL were averaged for each section. The 4 sections for each brain were then averaged to obtain per brain values. We analysed the data separately for dorsal (septo) and ventral (temporal) regions and infra- and supra-pyramidal blades of the GCL because these regions may have distinct functions [35] and differences in neurogenesis gradients [36]. Sections were considered ventral when the dentate gyrus in the ventral area of the brain section was obviously present, that is between -5.2 mm to -6.7 mm Bregma (Paxinos and Watson [37]) as previously done [38]. The percentage of GPER/Ki67 double-labelled cells was obtained from 15 brains (chosen randomly from all treatment groups) by randomly analyzing 100 Ki67-expressing cells in dorsal (50) and ventral sections (50) and determining whether those cells also expressed GPER (lack of co-labelling was also verified for some cells using confocal microscopy).

### Statistical analyses

All analyses were performed using Statistica v.8.0 (StatSoft Inc, Tulsa, OK) and using an α = 0.05. Normality and homogeneity of variance were verified using Kolmogorov-Smirnov and Brown-Forsythe tests respectively. Body mass was analyzed using one-way ANOVA with treatment group as the between-subject variable. Dentate gyrus volume and number of BrdU+ cells were each analyzed with repeated-measures ANOVAs with treatment group as the between-subjects factor and region (dorsal, ventral) and area (GCL, hilus) as the within-subjects factors. Differences in the number of BrdU+ cells between supra- and infra-pyramidal blades were analyzed using repeated-measures ANOVAs with treatment group as the between-subjects factor and regions (dorsal, ventral and supra-, infra-pyramidal blades) as the within-subjects factors. GPER optical density (OD) results were first analyzed using repeated-measures ANOVA with treatment group as the between-subjects factor and regions (dorsal and ventral and supra-, infra-pyramidal blades) as the within-subjects factors. As the main or interaction effects involving blade region were not significant, OD results were, therefore, analyzed using repeated-measures ANOVA with treatment group as the between-subjects factor and region (dorsal, ventral) and area (CA1, CA3) as the within-subjects factor. Post-hoc tests utilized Newman-Keuls. A priori comparisons were subject to Bonferroni corrections.

## Results

### Body mass and volume of the dentate gyrus did not significantly differ between groups

As expected, body mass did not significantly differ between treatment groups (all P’s>0.95; Table 1). For both experiments, there were no significant differences between treatment groups in the volume of the dentate gyrus (all P’s>0.28; Table 2). As expected, hilus volume was significantly larger than the GCL (Region effect: Experiment 1 F(1,19) = 716.65; P<0.001; Experiment 2 F(1,16) = 318.01; P<0.001) and the ventral region was significantly larger than the dorsal region (all P’s <0.001). We also analyzed the volume of the supra-pyramidal versus infra-pyramidal blades. The supra-pyramidal blade was significantly larger than the infra-pyramidal blade both in the dorsal and ventral regions (P<0.0001) but there were no significant main or interaction effects involving treatment (all P’s>0.19). Because overall there were no significant effects of treatment on volumes, we report the total number of BrdU+ cells.

**Table 1:** Mean ± SEM body mass of ovariectomized female rats the day of perfusion for Experiment 1 and 2.

Experiment	Treatment	Body mass (g)
1	Oil	296.00 ± 7.01
	E2	292.50 ± 8.59
	DMSO	293.83 ± 5.24
	G1-L	295.17 ± 3.32
	G1-M	296.50 ± 5.83
	G1-H	293.83 ± 3.51
2	Oil+DMSO	294.71 ± 3.51
	E2+DMSO	298.00 ± 7.75
	Oil+G15	294.50 ± 6.14
	E2+G15	293.86 ± 4.13

No significant effects of treatment on body mass were observed.

**Table 2:** Mean ± SEM volume of the granule cell layer (GCL) and the hilus (mm^3^) in female ovariectomized rats from Experiment 1 and 2 exposed to estradiol (E2), the GPER agonist G1, and the GPER antagonist G15.

Experiment	Treatment	N	Area	GCL (mm^3^)	Hilus (mm^3^)
1	Oil	4	Dorsal	1.28 ± 0.06	2.12 ± 0.14
			Ventral	1.59 ± 0.07	3.45 ± 0.27
	E2	5	Dorsal	1.39 ± 0.05	2.44 ± 0.05
			Ventral	1.86 ± 0.15	4.11 ± 0.31
	DMSO	6	Dorsal	1.31 ± 0.04	2.32 ± 0.06
			Ventral	1.83 ± 0.10	3.84 ± 0.19
	G1-L	2	Dorsal	1.25 ± 0.08	2.30 ± 0.22
			Ventral	1.70 ± 0.03	3.77 ± 0.22
	G1-M	4	Dorsal	1.10 ± 0.05	2.01 ± 0.06
			Ventral	1.79 ± 0.15	4.30 ± 0.30
	G1-H	4	Dorsal	1.37 ± 0.03	2.27 ± 0.12
			Ventral	1.89 ± 0.14	4.39 ± 0.41
2	Oil+DMSO	4	Dorsal	1.43 ± 0.11	2.14 ± 0.15
			Ventral	2.43 ± 0.22	4.02 ± 0.20
	E2+DMSO	5	Dorsal	1.45 ± 0.08	2.12 ± 0.18
			Ventral	2.46 ± 0.16	3.98 ± 0.44
	Oil+G15	5	Dorsal	1.52 ± 0.06	2.18 ± 0.07
			Ventral	2.26 ± 0.15	3.99 ± 0.20
	E2+G15	6	Dorsal	1.42 ± 0.06	2.05 ± 0.13
			Ventral	2.43 ± 0.13	4.18 ± 0.25

There were no significant differences between treatments observed in the volume of the GCL or hilus.

### Estradiol increases while the GPER agonist decreases cell proliferation in the dentate gyrus

In Experiment 1, estradiol increased the number of BrdU+ cells in the GCL relative to the oil control (P = 0.045) in the dentate gyrus but there were no other significant differences between groups (main treatment effect; F(4,16) = 3.14; P = 0.044; Fig 1C and 1D). As expected there were main effects of region and area (P<0.001) with the number of BrdU+ cells higher in ventral compared to dorsal regions and in the GCL compared to hilus. A priori, we thought there would be group differences between dorsal and ventral regions. A priori tests indicated that the GPER agonist, G1 at a medium dose (5 μg), decreased the number of BrdU+ cells in the dorsal but not ventral GCL relative to the DMSO control (P<0.001, P = 0.56, respectively; Fig 1C). No significant differences were detected for BrdU+ cells in the GCL between the high dose of G1 (10 μg) and DMSO (P>0.5). As indicated by the main treatment effect, estradiol increased the number of BrdU+ cells in both the dorsal and ventral regions (both P’s<0.002). DMSO alone increased the number of BrdU+ cells in the ventral, but not dorsal GCL, compared to the oil group (P = 0.003, P = 0.021 respectively, dorsal not significant due to Bonferroni correction). Unfortunately, tissue from four animals from the G1 low dose group (0.1 μg) could not be used for BrdU analysis. The two remaining animals from the G1 low group had a low number of BrdU+ cells (mean ± SEM = 2860 ± 760), a similar effect to the medium dose of G1 (3185 ± 435), however due to the low sample size, these animals were not included in the statistical analyses. Estradiol and G1 did not significantly affect the number of BrdU+ cells in the hilus (all P’s>0.15).

### Treatment with the GPER antagonist regionally affects cell proliferation in the dentate gyrus

In Experiment 2, treatment with estradiol+DMSO increased the number of BrdU+ cells relative to the oil+DMSO control in the dorsal GCL but not ventral GCL (P = 0.047, P = 0.21 respectively, Fig 1E and 1F, interaction effect: treatment by region (dorsal, ventral) by area (hilus, GCL); F(3,14) = 4.87; P = 0.016). Intriguingly, treatment with oil+G15 significantly increased the number of dorsal BrdU+ cells (P = 0.009), but decreased the number of ventral BrdU+ cells relative to oil+DMSO (P = 0.049; Fig 1F). The estradiol+G15 group did not significantly affect BrdU+ cells in the dorsal or ventral regions relative to oil+DMSO control (both P’s>0.5) or the estradiol+DMSO group (P = 0.07). There were no significant effects of treatment in the hilus (all P’s>0.7).

### Estradiol, G1, and G15 affected the number of BrdU+ cells equally in the infra- and supra-pyramidal blades

We analyzed differences in BrdU+ cell counts between the supra- and infra-pyramidal blades within the dorsal and ventral regions (Table 3). In Experiment 1, there was a significant effect of blade with the number of BrdU+ cells being significantly higher in the supra- compared to the infra-pyramidal blade in both the dorsal and ventral regions (F(1;17) = 12.18; P = 0.002). However, all treatments affected both blades equally (P>0.8). In Experiment 2, we did not find a blade effect (P>0.2) and all the treatments affected both blades equally in the dorsal and ventral regions (P>0.36).

**Table 3:** Mean ± SEM total number of BrdU+ cells in the infra- and supra-pyramidal blades in the dorsal and ventral regions of the granule cell layer (GCL) in female ovariectomized rats from Experiment 1 and 2.

Experiment	Treatment	Dorsal		Ventral	
		Infra GCL	Supra GCL	Infra GCL	Supra GCL
1	Oil	1785 ± 185.7	1965 ± 301.5	2140 ± 105.8	3905 ± 815.3
	E2	2910 ± 302.7	2490 ± 317.3	3690 ± 347.2	4630 ± 633.2
	DMSO	2683 ± 235.7	2593 ± 429.8	3220 ± 460.9	5057 ± 702.8
	G1-L	1330 ± 410.0	1530 ± 350.0	2680 ± 540.0	3310 ± 230.0
	G1-M	1560 ± 365.8	1625 ± 314.7	2955 ± 338.0	4245 ± 564.1
	G1-H	2245 ± 399.7	2310 ± 260.1	3065 ± 335.1	4535 ± 917.5
2	Oil+DMSO	2205 ± 125.5	1400 ± 63.77	3685 ± 277.1	4720 ± 259.7
	E2+DMSO	2728 ± 262.3	2128 ± 300.5	3168 ± 296.4	4616 ± 775.3
	oil+G15	2676 ± 212.8	2740 ± 402.3	3172 ± 383.2	3988 ± 340.7
	E2+G15	2605 ± 232.6	1320 ± 170.7	3970 ± 384.9	4730 ± 240.2

No significant effects of treatment were observed between the two blades.

### Expression of GPER in the dentate gyrus is decreased by G1 and increased by G15

We measured expression of GPER in the GCL, CA1, and CA3 regions using OD (Fig 2). In Experiment 1, there was a main treatment effect (F(5, 23) = 4.06; P = 0.009) but the effect of region and the interaction between region and treatment were not significant (P’s > 0.5) therefore data are presented as OD in the total (dorsal and ventral) GCL. Post-hoc tests revealed that G1 (5 and 10 μg) decreased GPER OD in the GCL relative to the DMSO control (P = 0.04 and P = 0.05, respectively; Fig 2E). Estradiol also showed a decreasing trend in GPER OD that was close to statistical significance (P = 0.07). Similarly, in Experiment 2 there was a main effect of treatment (F(3, 14) = 3.44; P = 0.046) regardless of region. Treatment with oil+G15 increased GPER OD relative to the oil+DMSO control (P = 0.02; Fig 2F). No other significant differences were detected. In the CA1 and CA3, GPER OD was not significantly affected by any of the treatments in Experiment 1 and 2, in either the dorsal or ventral regions (all P’s>0.7; Fig 2G and 2H). However, there was an area by region effect (F(1, 24) = 16.28; P<0.0004) and post-hoc tests revealed that GPER OD was significantly higher in the ventral relative to the dorsal region in both CA1 and CA3 regions and that the ventral GPER OD expression was higher in CA1 versus CA3 area (P = 0.0004).

### Ki67-expressing cells do not express GPER

We used double-label immunofluorescence for GPER and Ki67 to determine whether proliferating cells (Ki67-expressing cells) also express GPER. We found that GPER was expressed in the membrane of the granule cells of the GCL and very little expression was observed in the subgranular zone of the dentate gyrus (Fig 3). The percentage of Ki67-expressing cells co-localized with GPER was 0–1% and the treatments did not appreciably affect the co-localization percentage.

## Discussion

In the present study, we found that a high dose of estradiol increased cell proliferation in the dentate gyrus of the adult female rat, consistent with previous research [6,7]. We found that the GPER agonist G1 decreased cell proliferation, while the GPER antagonist G15 increased cell proliferation in the dorsal GCL. However, in the ventral GCL, G15 decreased cell proliferation and blocking GPER with G15 partially eliminated the estradiol-induced increase in cell proliferation in the dorsal GCL. G1 treatment decreased while G15 increased GPER expression in the dentate gyrus but not in the CA1 and CA3 region. Finally, Ki67-expressing cells in the dentate gyrus did not co-express GPER and treatment with estradiol, G1, or G15 did not affect co-localization of Ki67 with GPER, suggesting that the activation of GPER modulates cell proliferation via an indirect pathway. Thus, this study provides evidence that GPER has a novel role in regulating neurogenesis in the dentate gyrus in a regionally distinct manner and is partially independent of estradiol’s effects. Furthermore, our findings that G15 partially blocked estradiol’s ability to increase cell proliferation in the dorsal dentate gyrus coupled with our findings that G1 had opposing effects on cell proliferation than estradiol suggest that GPER may be partially involved in estradiol’s upregulation of cell proliferation.

### Estradiol increases, while activation of GPER decreases cell proliferation in the dentate gyrus

We found that estradiol increased cell proliferation in the dorsal and ventral dentate gyrus of adult female rats consistent with previous research using the same 10 μg dose, although previous research has not examined region-specific effects of estradiol [5,6,7,9,39]. Treatment with the GPER agonist G1 (5 μg) decreased, while treatment with the antagonist G15 (40 μg) increased cell proliferation in the dorsal dentate gyrus. Interestingly, treatment with G15 resulted in the opposite effect on cell proliferation in the ventral region. When given in combination with estradiol, G15 partially blocked the estradiol-induced increase in cell proliferation in the dorsal GCL. At high doses (>10 μM) G15 can bind weakly to ERα and ERβ [40], although chronic treatment with 40 μg of G15 blocked the modulation of hippocampus-dependent memory by estradiol [20]. Thus, it is possible that in our study the increase in cell proliferation in the dorsal region observed with G15 was due to non-specific activation of ERs by G15, however this does not explain the decrease in cell proliferation with G15 in the ventral region and the attenuation of cell proliferation by E2+G15 in the present study. Previously, we found that ERα and ERβ agonists (using PPT and DPN respectively) increase cell proliferation but not to the same extent as estradiol alone [12] and the ER antagonist ICI 182,780 only partially blocked the effects of estradiol [13]. Together with the present study, these results suggest that other receptor/mechanisms are at play in the effect of estradiol to upregulate cell proliferation. It is possible that membrane localized ERα and ERβ mediate some of the effects of estradiol on cell proliferation. A membrane bound ERα was implicated in regulating hippocampal memory by interacting with the glutamate receptor (mGluR1a) [41] supporting the idea that estradiol can have multiple mechanisms of action in the hippocampus. Other estrogen receptors have been identified (ER-X, Gq-mER; [42,43]) and 17α-estradiol, a putative ligand of ER-X, increases cell proliferation in the dentate gyrus [6] but the role of Gq-mER in adult neurogenesis remains to be determined.

Neurogenesis is a multi-stage process including cell proliferation, migration, differentiation, and survival. A net increase or decrease in new neurons is determined by changes in any one of these components independently or in concert (reviewed in [5]). In adult rats, the majority of new cells in the dentate gyrus do indeed become neurons (~75–85%; e.g., see [44,45]). Thus, we expect that initial increases in cell proliferation would result in an increase in the number of new neurons. In this study, we examined effects of G1 and G15 at only one time point, and because the effects of estradiol are time-dependent [9,39], it would be interesting to test whether G1 and G15 have different effects on cell proliferation at different time points.

Although GPER has been accepted as an estrogen receptor, some controversies still surround this receptor, including specificity of the ligand and whether this receptor is exclusively an estrogen receptor [46,47]. G1 and G15 were designed to specifically act on GPER [21,48] and these compounds have been used to demonstrate that actions of estradiol are mediated by this receptor (e.g., [18,20,49,50]). Nevertheless, studies have questioned whether estradiol is the unique ligand for GPER since aldosterone can also activate GPER in some tissues such as coronary microarteries and muscle cells [51,52,53]. In the present study, the GPER agonist decreased cell proliferation and aldosterone also decreases cell proliferation in male rats [54]. However, activation of GPER by other ligands has not been shown in the brain and remains controversial as Cheng et al. found that aldosterone does not bind to GPER in the kidney [55]. In addition, the ERα agonist PPT can also activate GPER in mouse uterine epithelial cells [56] indicating that the current pharmacological ER and GPER modulators may not be specific. Thus clearly more work needs to be done to determine the ligands and specificity of these ligands to GPER in the hippocampus.

### GPER expression is regulated by G1 and G15 in the dentate gyrus

We found that GPER is expressed in the granule cells of the dentate gyrus and in pyramidal neurons of the CA1 and CA3 regions of adult ovariectomized female rats, consistent with previous studies in intact male and female rats and mice [15,16,17]. In the current study, we showed for the first time that GPER expression was downregulated by G1 and estradiol (trend for statistical significance) while G15 increased expression of GPER in the dentate gyrus but not in the CA1 and CA3 regions. Thus, in contrast to the effects of estradiol and G1 to upregulate and downregulate cell proliferation, respectively, both estradiol and G1 treatments resulted in a downregulation of GPER expression in the GCL. This pattern of results suggests that this negative autoregulation of GPER expression in the dentate gyrus is not involved in the regulation of cell proliferation. Treatment with the antagonist G15 alone resulted in the opposite effect, upregulating GPER expression in the dentate gyrus; however, when G15 was administered with estradiol it did not significantly affect the expression of GPER relative to G15 and estradiol treatments alone. These results may indicate that G15 regulates GPER by blocking the binding of a ligand other than estradiol.

To our knowledge, we showed for the first time that estradiol did not affect GPER expression in the CA1 and CA3 regions of the hippocampus. This finding is consistent with previous research that expression of GPER is not regulated by the estrous cycle phase or ovariectomy in the CA1 and CA3 of adult female rodents. Broughton et al. [57] found similar levels of GPER (assessed by semi-quantitative analysis) in the CA regions between intact and ovariectomized female mice. Using immunoblot analysis of the whole hippocampus, Matsuda et al. [17] found that GPER expression did not vary between adult females in proestrus or estrus and male rats. Other brain regions (nucleus of the solitary tract, periaqueductal gray, ventrolateral medulla, paraventricular nucleus of the hypothalamus) show significant variations in GPER mRNA during the estrous cycle and after ovariectomy in rats [58]. Together with the present study, these studies suggest that estradiol tended to regulate GPER expression in the dentate gyrus and other brain regions, but not in Ammon’s horn.

### Not all vehicles are created equally

In Experiment 1, we found that estradiol dissolved in oil significantly increased cell proliferation relative to the oil group in both the dorsal and ventral regions and DMSO increased cell proliferation relative to oil controls in the ventral region. In Experiment 2 however, we found that estradiol+DMSO increased cell proliferation relative to oil+DMSO group only in the dorsal region. These experiments indicate that DMSO increases ventral cell proliferation on its own and that DMSO may interfere with estradiol’s ability to increase cell proliferation in the ventral GCL. However, it is important to note that 17β-estradiol, whether using oil, DMSO or both as a vehicle increased cell proliferation compared to the appropriate control in both Experiment 1 and 2. Taken together, these results indicate that DMSO may have independent effects on the ventral hippocampus that result in changes in neuroplasticity. Based on our results, we advise caution when using DMSO as a vehicle.

### Regional differences in the regulation of cell proliferation

The effects of G1 on cell proliferation and GPER expression in the GCL were preferentially observed in the dorsal region of the dentate gyrus. In addition, the effects of G15 differed depending on the region: increasing and decreasing cell proliferation in the dorsal and ventral regions respectively. These findings are intriguing as there are dorsal/ventral differences in biological function of the hippocampus. The dorsal region is associated with cognition and reference memory while the ventral region is associated with stress, emotion, and working memory [35,59,60]. In fact high estradiol (10 μg) impairs spatial reference memory [25] which implicates the dorsal region of the hippocampus [59]. While, low (0.32 μg) and medium (1 and 5 μg) levels of estradiol facilitate and impair spatial working memory, respectively [61]. On the other hand, adrenal steroids are involved in regulating both stress and cognition and can interact with estradiol to regulate both cell proliferation [9] and cognition [62,63,64,65]. Activation of GPER with G1 (5 μg) positively regulates spatial working memory in female rats [18]. Increases in cell proliferation have been linked to poorer performance on hippocampus-dependent tasks (reviewed in [5,66]) and computer modelling also suggests that cell proliferation would interfere with learning and memory [67]. Further experiments could examine the influence of chronic exposure of GPER agonists on survival of new neurons that have been incorporated into the existing circuitry and may therefore have more of an influence on function.

Even though, the effects of G1 and G15 are mostly in the dorsal region, this regional difference is not related to gradients in ER or GPER expression in the dentate gyrus. Our OD results indicate a similar expression in the dorsal and ventral portions of the dentate gyrus, although we observed higher expression of GPER in the ventral relative to the dorsal regions in the CA1 and CA3 areas. Expression of other components of the transcription/translation machinery may shed some light into this regional difference.

### GPER is not expressed in proliferating cells

Our results indicate that Ki67-expressing cells do not co-express GPER in the adult female Sprague-Dawley rat. This suggests that the mechanism of G1 and G15 to alter cell proliferation is via an indirect mechanism. This is similar to the expression of nuclear ERs in adult female Sprague-Dawley rats. Mazzucco et al., showed a low percentage of Ki67-expressing cells co-localize with ERα (between 3–7%) and ERβ (between 3–11%) mRNA and treatment with estradiol or ER agonists (PPT, DPN) do not affect co-localization [12]. In contrast, the number of BrdU+ cells (after 4 injections of BrdU, given 6 h apart, such that BrdU+ cells would be a mixture of dividing progenitor and resulting daughter cells) in the dentate gyrus that express ERα is higher at around 43% in female Meadow voles and 80% in female Prairie voles [68]. Furthermore, in male Sprague-Dawley rats approximately 80% of Ki67-expressing cells co-expressed either ERα or ERβ in the subgranular zone [69]. These data suggest great variability, dependent on sex and species, of ER expression in dividing cells. According to these studies, estradiol could directly act via ERs in proliferating cells to modulate neurogenesis but any potential ligand of GPER does not act directly on proliferating cells as we found no evidence of GPER co-localization with Ki67- expressing cells. This suggests an indirect mechanism of GPER agonists and antagonists to modulate cell proliferation in the hippocampus.

## Conclusions

Activation of GPER decreased cell proliferation in the dorsal hippocampus, while inactivation of GPER increased or decreased cell proliferation depending on the region of the dentate gyrus in female rats. We found no evidence of GPER localization on dividing cells in the dentate gyrus suggesting that the effects of GPER agonists and antagonists to modulate cell proliferation are indirect. These findings present a regulatory role for GPER on neuroplasticity, independent of estradiol’s effects, in the adult female rat hippocampus. Further, we found that the GPER antagonist partially blocked the estradiol-induced increase in cell proliferation in the dorsal dentate gyrus. Intriguingly, estradiol and G1 decreased GPER expression in the dentate gyrus but not the CA1 and CA3, further suggesting that GPER activation by G1 regulates cell proliferation independent of estradiol. Finally, we present here a novel role for GPER in regulating neurogenesis in the hippocampus and suggest novel pathways for neuroplasticity.
